# Adipose-derived mesenchymal stem cells from liposuction and resected fat are feasible sources for regenerative medicine

**DOI:** 10.1186/s40001-017-0258-9

**Published:** 2017-05-19

**Authors:** Sandra Schneider, Marina Unger, Martijn van Griensven, Elizabeth R. Balmayor

**Affiliations:** Experimental Trauma Surgery, Department of Trauma Surgery, Klinikum rechts der Isar, Technical University of Munich, Ismaninger Strasse 22, 81675 Munich, Germany

**Keywords:** Adipose tissue, Mesenchymal stem cells, Liposuction, Solid fat, Growth potential, Surface characterization

## Abstract

**Background:**

The use of mesenchymal stem cells (MSCs) in research and in regenerative medicine has progressed. Bone marrow as a source has drawbacks because of subsequent morbidities. An easily accessible and valuable source is adipose tissue. This type of tissue contains a high number of MSCs, and obtaining higher quantities of tissue is more feasible. Fat tissue can be harvested using different methods such as liposuction and resection. First, a detailed isolation protocol with complete characterization is described. This also includes highlighting problems and pitfalls. Furthermore, some comparisons of these different harvesting methods exist. However, the later characterization of the cells is conducted poorly in most cases.

**Methods:**

We performed an in-depth characterization over five passages including an investigation of the effect of freezing and thawing. Characterization was performed using flow cytometry with CD markers, metabolic activity with Alamar Blue, growth potential in between passages, and cytoskeleton staining.

**Results:**

Our results show that the cells isolated with distinct isolation methods (solid versus liposuction “liquid”) have the same MSC potential. However, the percentage of cells positive for the markers CD73, CD90, and CD105 is initially quite low. The cells isolated from the liquid fat tissue grow faster at higher passages, and significantly more cells display MSC markers.

**Conclusion:**

In summary, we show a simple and efficient method to isolate adipose-derived mesenchymal stem cells from different preparations. Liposuctions and resection can be used, whereas liposuction has more growth potential at higher passages.

## Background

The use of mesenchymal stem cells (MSCs) in research and clinical applications has progressed during the last decades. Hence, it has become even more important to take a closer look and specify feasible sources. In principle, MSCs can be obtained from many different types of tissue like bone marrow, adipose tissue, synovium, dermis, peripheral blood, umbilical cord, and placenta [1–3]. Until now, bone marrow (BM) has been most commonly used to isolate MSCs for further use, but it has some considerable, negative issues for the patient and also some disadvantages compared to other tissue types. Harvesting of bone marrow from, e.g. the iliac crest is an invasive procedure. It is accompanied with severe pain and a high risk of infection. The collectable volume of bone marrow is low and highly limited, resulting in a low yield of isolatable cells [4]. In comparison, adipose tissue shows some promising benefits. It can be easily collected in higher amounts and from different parts of the human body. The harvesting procedure is often part of a different operation and is not necessarily the only reason for the intervention. Most of the obtainable tissue is ethically uncontroversial as it is waste material resulting from the operation. This is for instance the case for liquid fat after a liposuction or solid fat after an abdominoplasty. As adipose-derived mesenchymal stem cells (AMSCs) are a very promising approach for the future and they can be obtained from different sources, there is a need to compare the different adipose tissue localizations to characterize the possible attributes/properties. In the literature, direct comparisons of these different harvesting techniques are sparse. Faustini et al. [5] investigated liposuction versus resection by morphological differences. A similar study was performed by Eto et al. [6]. Both studies, however, did not further or fully characterize the obtained cells. There was only a morphological characterization or some more characterizations, but not for the positive markers of stem cells [5–7]. Other papers deal with different localizations, but do not differentiate between the harvesting method used [8–10]. In general, MSCs are defined by specific properties, such as adherence on plastic and colony formation capability. They express a specific pattern of CD markers [11, 12]. MSCs express CD73, CD90, and CD105 and have a lack of expression regarding the hematopoietic progenitor cell antigen CD34, the leukocyte antigen CD45, the monocyte marker CD14, and lymphocyte marker CD20. Furthermore, they can be differentiated into at least three different tissues [12].

Others compared different liposuction methods, automated machines and/or AMSC isolation methods [13–22]. Thus, liposuction versus resection have hardly been compared directly and we aimed to perform this in our study. Moreover, we wanted to provide a detailed protocol for obtaining AMSCs.

## Methods

### Materials and reagents

Cell culture medium (Dulbecco’s modified eagle medium, 4500 mg/l glucose, 0.584 g/l l-glutamine, DMEM), fetal bovine serum (FBS), penicillin/streptomycin (P/S), trypsin–EDTA, and Dulbecco’s phosphate buffered saline (DPBS) were purchased from Sigma-Aldrich (St. Louis, Missouri, USA). Collagenase Type II and trypan blue were purchased from Biochrom/Merck (Darmstadt, Germany). Dimethyl sulfoxide (DMSO) was purchased from Carl Roth (Karlsruhe, Germany). The cell culture reaction tubes and cell culture flasks T175 were purchased from Eppendorf AG (Hamburg, Germany). Sterile cell strainer (40 µm) was purchased from Corning Inc. (Corning, New York, USA). The freezing containers used for cryopreservation were Nalgene^®^ Mr. Frosty purchased from Sigma-Aldrich (St. Louis, Missouri, USA).

### Human tissue

Fresh adipose tissue from patients between 30 and 78 years old (58 ± 21.1; *n* = 6), who underwent liposuction (*n* = 3; 40 ± 11.1 years) or resection during implantation of a hip endoprosthesis (*n* = 3; 76 ± 3.5 years), was used. Patients provided informed consent. The ethical committee of the university hospital “Klinikum rechts der Isar” of the Technical University of Munich granted permission to collect the tissue. All procedures were carried out in accordance with the declaration of Helsinki in its latest amendment.

### Adipose-derived mesenchymal stem cell isolation

**Fig. 1:**
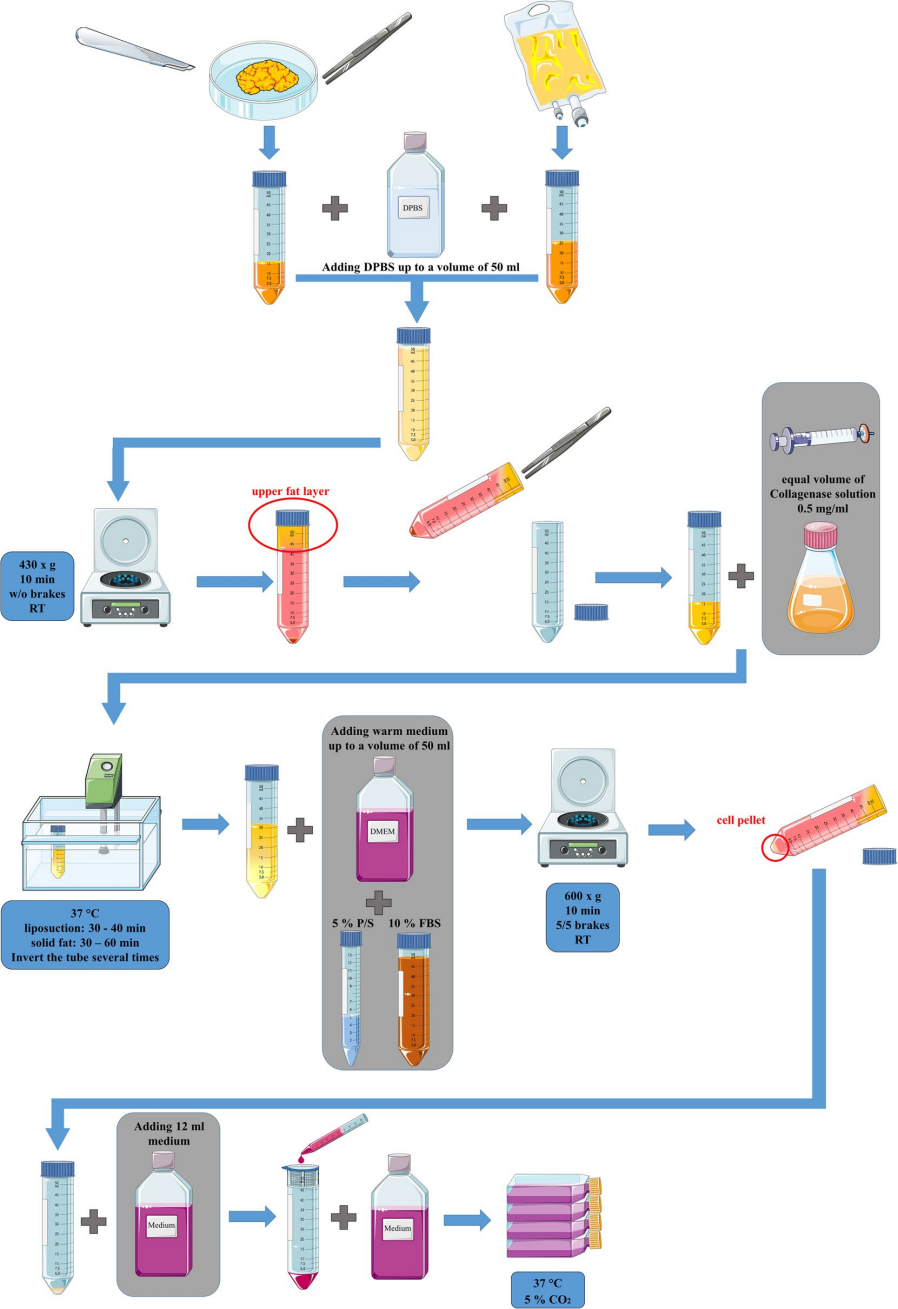
Schematic overview of the different isolation steps for adipose tissue in solid state and liposuction (*P/S* penicillin/streptomycin, *FBS* fetal bovine serum, *DPBS* Dulbecco’s phosphate buffered saline, *RT* room temperature, *w/o* without)

**Fig. 2:**
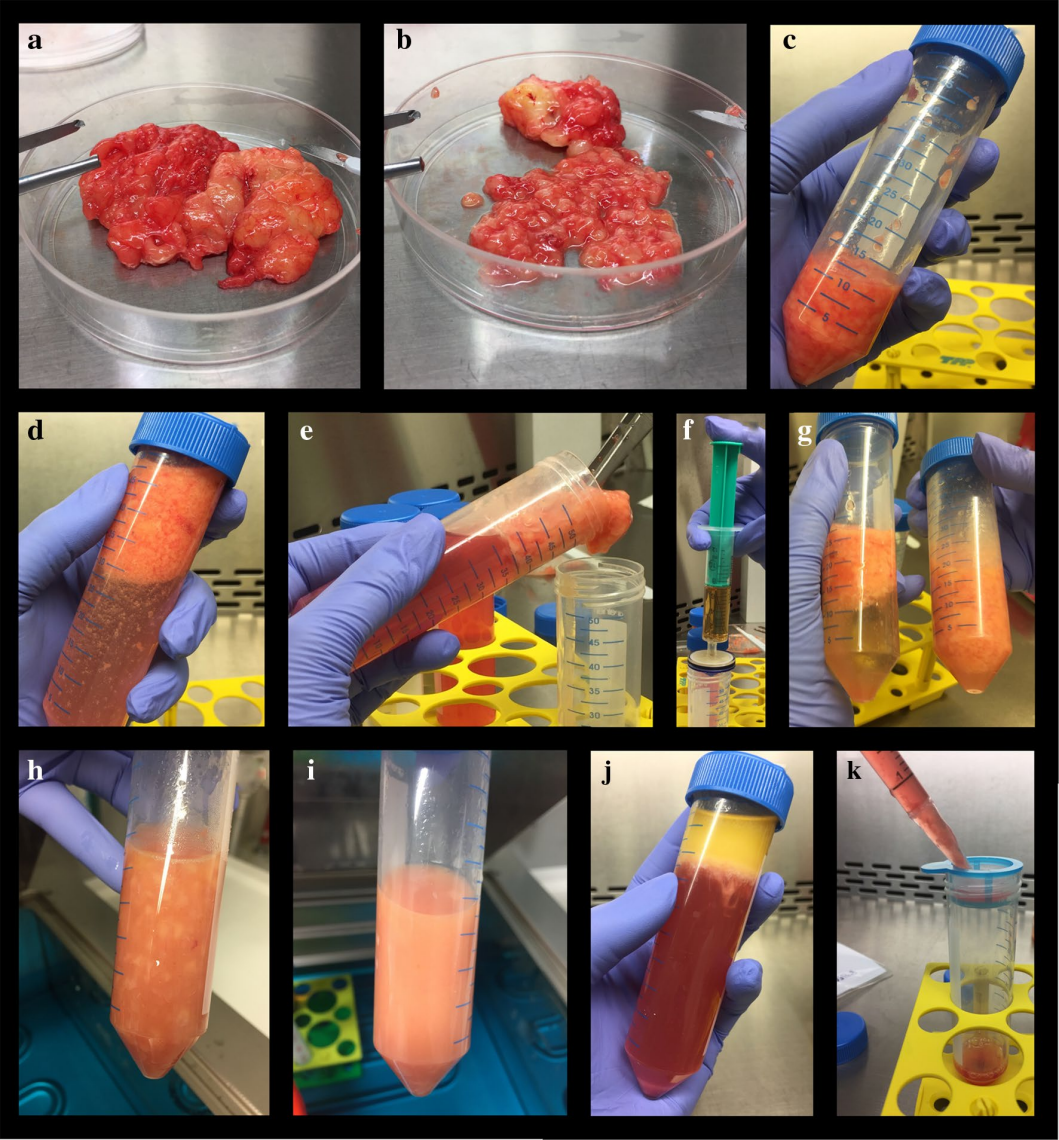
Photographs of the important steps of the isolation process **a** solid fat from abdominoplasty **b** solid fat cut into small pieces (at a range of 1–3 mm) by using a scalpel and forceps **c** transferring 10–15 ml of minced fat into a 50 ml reaction tube **d** adding DPBS up to a total volume of 50 ml **e** transferring of the upper fat layer into a new reaction tube **f** adding an equal volume of collagenase solution (0.5 mg/ml in DPBS; 355 U/mg) through a filter **g** mixing the minced fat with the collagenase solution **h** appearance of the mixture of fat tissue and collagenase just before incubation at 37 °C with pieces of fat tissue visible **i** homogeneous emulsion without remaining pieces of fat tissue after incubation at 37 °C in a water bath **j** discarding the upper fat layer and removing the supernatant after centrifugation **k** filtrating the suspension through a cell strainer (40 µm) into a fresh reaction tube

**Fig. 3:**
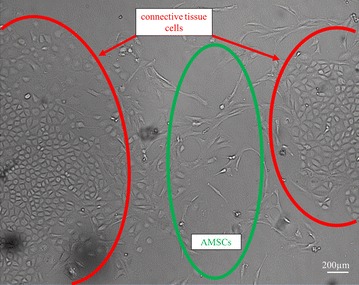
Adipose-derived mesenchymal stem cells (AMSCs) with contamination of connective tissue cells (bright field, 40× magnification)

For the isolation of AMSCs (Fig. 1), the first step of the procedure differs from solid fat (Fig. 2a) to liposuction. Due to the relatively small effective surface area in the solid adipose tissue compared to the already processed liposuction material (minced during the harvesting procedure), an additional step of surface enlargement is necessary. Therefore, the solid fat was cut into small pieces (at a range of 1–3 mm) (Fig. 2b) by using a scalpel and forceps. Upon completion, 10–15 ml of minced fat were transferred into a 50 ml reaction tube (Fig. 2c). From the liposuction material, 25 ml fat was transferred into a 50 ml reaction tube. Note that, a transfer of connective tissue should be avoided as the possibility of contamination with connective tissue cells increases (Fig. 3).

For all samples, it is mandatory that they are immediately isolated, as cell death will rapidly occur considering insufficient nourishment of the tissue after removal from the living body.

To remove remaining blood from the tissue as it interferes with enzymatic digestion, DPBS was added up to a total volume of 50 ml (Fig. 2d). The fat was washed by centrifugation (430×*g* 10 min, RT, w/o brakes). The upper fat layer (Fig. 2e) was transferred into a new reaction tube. If the fat tissue still appeared reddish, the washing step was repeated. Next, an equal volume of collagenase solution (0.5 mg/ml in DPBS; 355 U/mg) was added and the mixture was incubated at 37 °C in a water bath (Fig. 2f, g).

In order to evenly distribute the floating fat in the collagenase solution, the reaction tube was inverted several times every 10 min. To determine the completion of the digestion, it is important that the fat-collagenase mixture is homogenous. The mix should have turned into a homogeneous emulsion without remaining pieces of fat tissue (Fig. 2h, i). The period after which the digestion should be stopped depends on the type of tissue. The typical digestion time for a liposuction is 30–40 min as the overall time for the digestion of solid fat tissue needs to be extended up to 1 h. Over-digestion of the material needs to be avoided in order to prevent harming the MSCs. To inhibit the enzymatic activity, warm culture medium (DMEM supplemented with 5% P/S and 10% FBS) was added up to a total volume of 50 ml and mixed carefully. After centrifugation (600×*g*, 10 min, RT, brakes 5/5) (Fig. 2j), the upper fat layer was discarded and the supernatant was removed. The remaining cell pellet containing the MSCs was resuspended with 12 ml of culture medium. To get rid of remaining tissue parts, the suspension was filtrated through a cell strainer (40 µm) into a fresh reaction tube (Fig. 2k). After adding 15 ml warm culture medium, the cell suspension was transferred into a T175 culture flask. The cells were incubated in a humidified environment at 37 °C and 5% CO_2_. The dimension of the growth surface depends on the initial volume of fat and the donor variability. However, cell counting in the suspension is hardly possible at this stage. Therefore, the recommended ratio for solid adipose tissue is 175 cm^2^/10–15 ml of fat and for a liposuction 175 cm^2^/25 ml of adipose tissue.

In order to remove non-adherent cells, the culture medium was changed after 1 day of cultivation. Before aspirating the culture medium, the flask was gently shaken to stir up non-adherent cells. 20 ml of DPBS was added to remove all residues of blood and unwanted connective tissue cells. The flask was again gently shaken and the DPBS was aspirated. Warm culture medium was added to the cells (25 ml/175 cm^2^) and they were incubated for further culture at 37 °C and 5% CO_2_. A medium change was performed twice a week.

#### Expansion

After reaching a confluence of 80–90%, the cells were subcultured into new flasks. The supernatant was aspirated and the cells were washed with 15 ml DPBS in order to remove interfering proteins. Then, trypsin/EDTA solution was added (2 ml/T175 flask) and dispensed thoroughly. After 5 min at 37 °C/5% CO_2_, detachment of the cells was checked under the microscope. The cells appeared round-shaped and were already partially detached from the surface. Providing mechanical force by hitting the flask with the palm of the hand several times completed the detachment process. To stop the enzyme activity, 15 ml culture medium was added. The growth surface was rinsed with the medium, which was transferred to a 50 ml tube. The suspension was centrifuged at 500×*g* for 10 min at RT (with brakes) and the supernatant was aspirated. The cell pellet was resuspended in warm culture medium and seeded into new cell culture flasks. A recommended splitting ratio is 1:3–1:4, meaning the expansion of the growth surface from 1 × 175 cm^2^ to 3 × 175 cm^2^ or 4 × 175 cm^2^.

#### Freezing

To use cells at a later time without wanting them to age and lose their stem cell properties, they must be frozen. For the later described methods, cells were frozen in passage 1. Therefore, the cells in passage 1 were detached from the cell culture flask—as already described—and the total cell amount was obtained by counting the cells using the trypan blue exclusion method. To adjust the right volume of cells, they were centrifuged again and the supernatant was aspirated. The required volume of freezing medium containing 50% cell culture medium, 40% FCS and 10% DMSO, as freezing agent, was prepared, cooled down, and then added to the cells obtaining a cell density of 1–2 × 10^6^ cells/ml. The cell suspension was distributed into freezing tubes, which were immediately put into a freezing container with isopropanol (Mr. Frosty) providing a consistent freezing rate of 1 °C/min when stored at −80 °C. After 1 day at −80 °C, the tubes were transferred to a nitrogen tank for long-term storage.

#### Thawing

To use the cells for further experiments, they were thawed. Therefore, 40 ml warm culture medium was added into a 50 ml reaction tube. Cells were thawed by adding warm medium rapidly to the freezing tube and transferring the cells into a 50 ml reaction tube. After centrifugation at 300×*g* for 10 min, the supernatant was aspirated; the cell pellet was resuspended in culture medium and transferred into flasks (1 × 10^6^ cells/175 cm^2^). After 24 h, the attachment of cells was checked and the medium was changed to remove dead cells.

### Flow cytometry

The cells from the different donors were used to characterize them regarding MSC properties. This was done with the isolated cells after freezing (frozen in passage 1) and over different passages up to passage 6. Flow cytometry data were obtained using a MACSQuant Analyzer (Miltenyi Biotech GmbH, Bergisch Gladbach, Germany). The MSC Phenotyping Kit (Miltenyi Biotec GmbH, Cat #130-095-198) was used according to the manufacturer’s information to obtain data of expression of CD73 (APC-conjugated), CD90 (FITC-conjugated), CD105 (PE-conjugated), CD14, CD20, CD34, and CD45 (all PerCP-conjugated). After 72 h of culture at 37 °C/5%CO_2_, cells were detached by adding trypsin/EDTA, counted and stained with 10 µl of the MSC Phenotyping Cocktail and the respective Isotype Control Cocktail. At least 30,000 events were measured. The remaining unstained cells were seeded on flasks (0.6 × 10^4^ cells/cm^2^) to perform this same analysis at a higher passage. The data analysis was performed with the software FlowJo (FlowJo LLC, Ashland, Oregon, US). Gates were set first based on the granularity and size of the cells of interest with the help of side and forward scatter. Subsequently, the cells from this gate were shown in the respective fluorescence channels based on the fluorochrome attached.

### Alamar Blue

To determine the metabolic activity of the cells, Alamar Blue assay was performed. Therefore, the cells were seeded in triplicates in a 48-well plate. After an adjustment period, 1/10 of the total volume Alamar Blue reagent (Biozol, Eching, Germany, BZL00727) was added to each well and the fluorescence signal was measured after 1, 2, 3, 4, and 5 h with FLUOStar Omega (BMG labtech, Ortenberg, Germany) at 570 nm and 600 nm.

### Growth potential

For comparison of the growth potential from solid and liquid fat tissue, the cell number was counted in each passage up to passage 5. For this, cells were seeded with a density of 0.6 × 10^4^ cells/cm^2^ and cultured for 3 days at 37 °C and 5% CO_2_. Thereafter, the cells were enzymatically detached by trypsin/EDTA and counted with the trypan blue exclusion method. Subsequently, they were seeded with a density of 0.6 × 10^4^ cells/cm^2^ and further cultured at a humidified environment.

### Cytoskeleton staining

To observe the cytoskeleton of the cells over the different passages, Phalloidin/Hoechst staining was performed. Therefore, cells were seeded (0.6 × 10^4^ cells/cm^2^) in a 48-well plate. The cells were washed with DPBS and fixed with 3.7% formaldehyde (Carl Roth, Karlsruhe, Germany, 7398.1, CAS 50-00-0) for 30 min. For permeabilization, the cells were washed twice and 0.2% TritonX-100/DPBS (Carl Roth, Karlsruhe, Germany, 3051.2, CAS 002-93-1) was added for 5 min and washed three times with DPBS. The cells were stained with 0.5 µg/ml Phalloidin solution (Sigma-Aldrich, St. Louis, Missouri, USA, P2141, CAS 17466-45-4) for 40 min and 4 µg/ml Hoechst (Sigma-Aldrich, St. Louis, Missouri, USA, 14533, CAS 23491-52-3) was added for 5 min. The pictures were taken with the microscope BZ-9000 from Keyence (Osaka, Japan) in a magnification of 200× from cells immediately after thawing and in passage five.

### Statistical analysis

Statistical analysis was performed using GraphPad Prism (GraphPad Software, CA, USA). All values are reported as mean ± standard deviation. Normality of the data was confirmed by D’Agostino-Pearson test. Probabilities of *P* < 0.05 were considered as significant. Flow cytometry analysis of the single stained cells was performed for every CD marker over the passages with one-way ANOVA followed by a post hoc Tukey’s multiple comparison test. For comparing the results of the isolated cells from the solid fat tissue with the cells from the liquid fat tissue, two-way ANOVA (Bonferroni’s multiple comparisons test) was used. To compare the cells of both fat tissue types, which were CD73^+^, CD90^+^, and CD105^+^, multiple *t* test was used. For comparing cells over passages, a Kruskal–Wallis test was performed. For the metabolic activity, the slopes of the regression curves were compared. Growth potential analysis was performed with Mann–Whitney-*U*-test for the comparison between solid and liquid cell sources, and Kruskal–Wallis-test was used for comparing the individual respective cell sources over the passages.

## Results

**Fig. 4:**
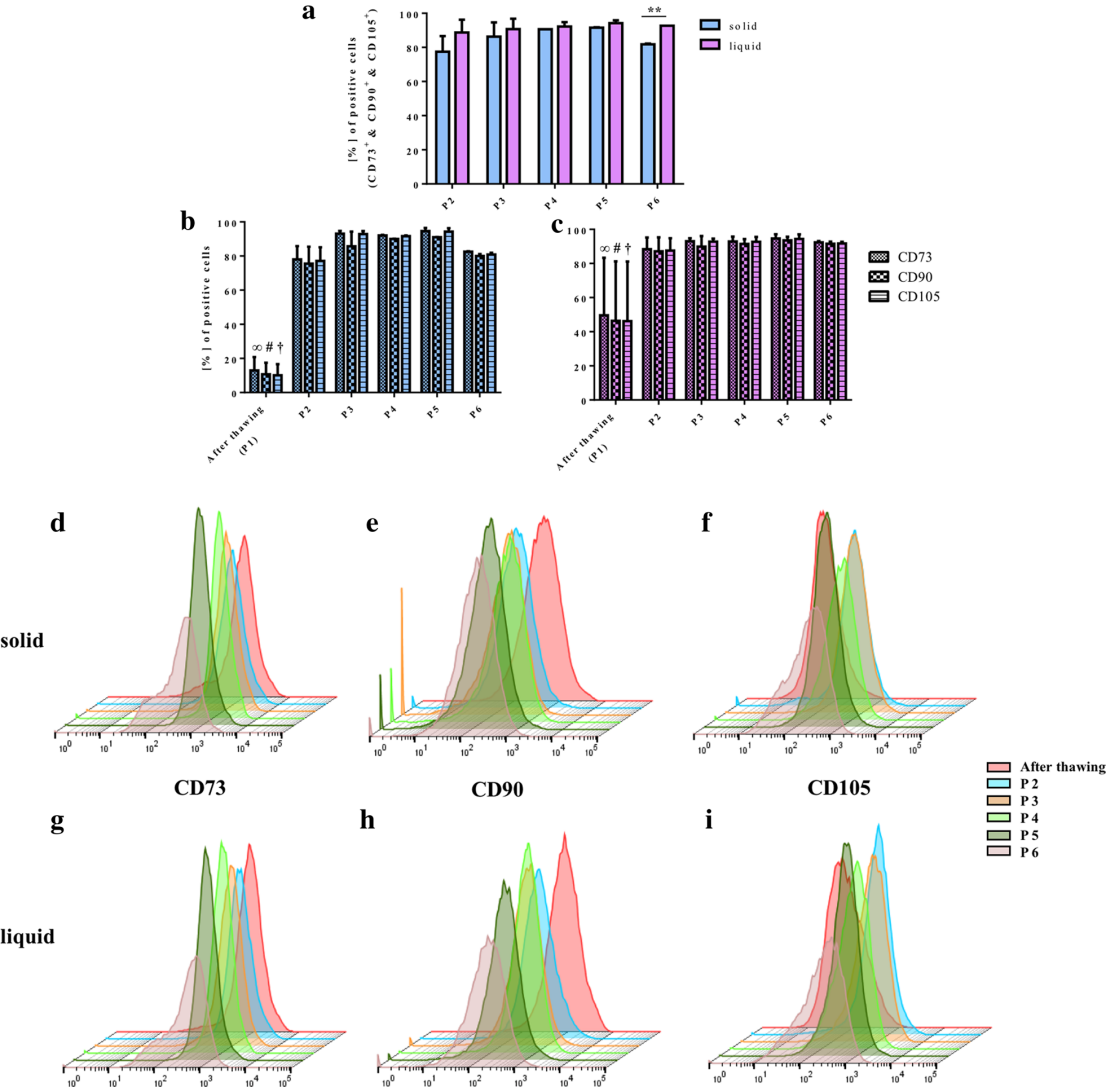
Flow cytometry: percentage of CD73^+^, CD90^+^, and CD105^+^ cells (**a**), percentages of each separate marker in each passage for cells from solid fat (**b**) and from liquid fat (**c**), example of a staged histogram with a 3D presentation per marker over the different passages for CD73^+^ cells from solid fat (**d**) and from liquid fat (**g**), CD90^+^ cells from solid fat (**e**) and from liquid fat (**h**), CD105^+^ cells from solid fat (**f**) and from liquid fat (**i**) (*infinity*, *hash*, *dagger*: the percentage of positive cells after thawing is significantly lower in comparison to every other passage for the various CD markers)

Cells harvested with the two different methods were analyzed for the expression of the typical MSC markers, CD73, CD90, and CD105 (Fig. 4). All cell populations in passage two to six were negative for CD14, CD20, CD34, and CD45. Cells isolated from solid fat tissue, which were directly analyzed after thawing, were only 10% positive for CD90 or CD105. Concerning CD73, there were 13% cells positive (Fig. 4b). In comparison, cells isolated from the liquid fat tissue had significantly higher percentages of CD90^+^ or CD105^+^ cells (46%; *P* < 0.05; *P* < 0.01) and for CD73^+^ cells (50%; *P* < 0.01) at that time (Fig. 4c). In contrast, in higher passages there was no significance between the positively stained cells from solid fat tissue to the positively stained cells from liquid fat tissue for every single marker. The percentage of positive cells in passage two to six from the solid fat tissue were significantly higher for every marker compared to the cells directly after thawing (*P* < 0.001). This was also the case for the isolated cells from liquid fat tissue for the CD73 and CD105 markers (*P* < 0.05). The cells, which were isolated from liquid fat tissue, in passages three to six had significantly higher expression of CD90 as compared to the cells after thawing (*P* < 0.05). There was no difference between the histogram profiles of the cells from the solid fat tissue compared to the cells from the liquid fat tissue (Fig. 4d, e). The percentages of CD73^+^, CD90^+^, and CD105^+^ cells of every cell population from passage two to five were not significantly different when the cells from the solid fat tissue isolation were compared to the cells from the liquid fat tissue isolation (Fig. 4a). The CD73^+^, CD90^+^, and CD105^+^ cell population from the liquid fat tissue isolation were significantly higher (*P* < 0.01) in passage six than the cells population from the solid fat tissue isolation.

**Fig. 5:**
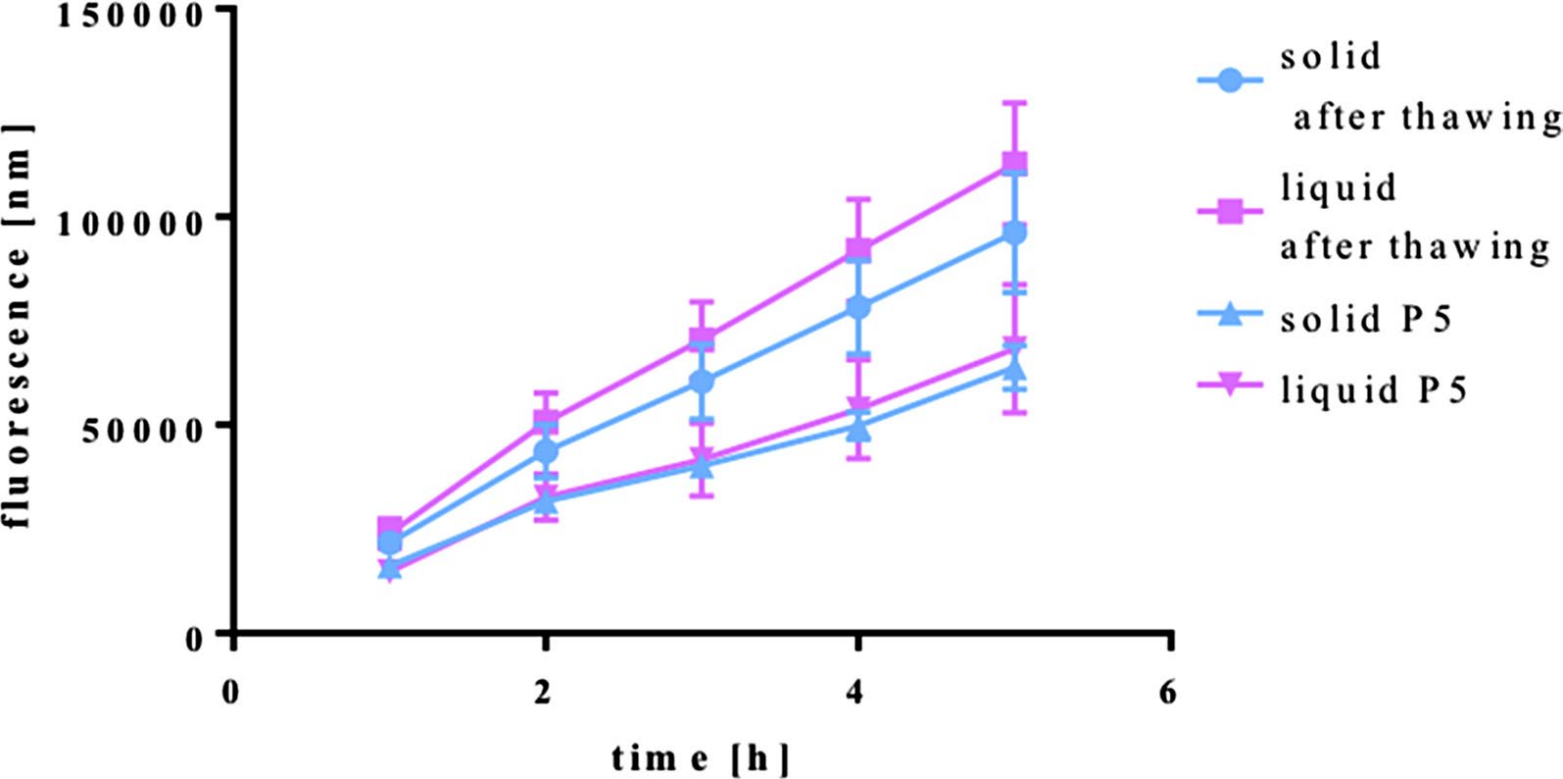
Metabolic activity (Alamar Blue) measured over a 5 h period

The metabolic activity was measured at two time points. A measurement at an early time point was performed in cells directly after thawing. The latest measurement was conducted with cells in passage five (Fig. 5). The slopes of the curves isolated from both harvesting methods were extremely significantly different over the different passages (*P* < 0.0001). The curves of the thawed cells isolated from the solid fat tissue were significantly lower than those from the thawed cells isolated from the liquid fat tissue (*P* < 0.05). The slopes of the curves of those cells at passage five were not significantly different.

**Fig. 6:**
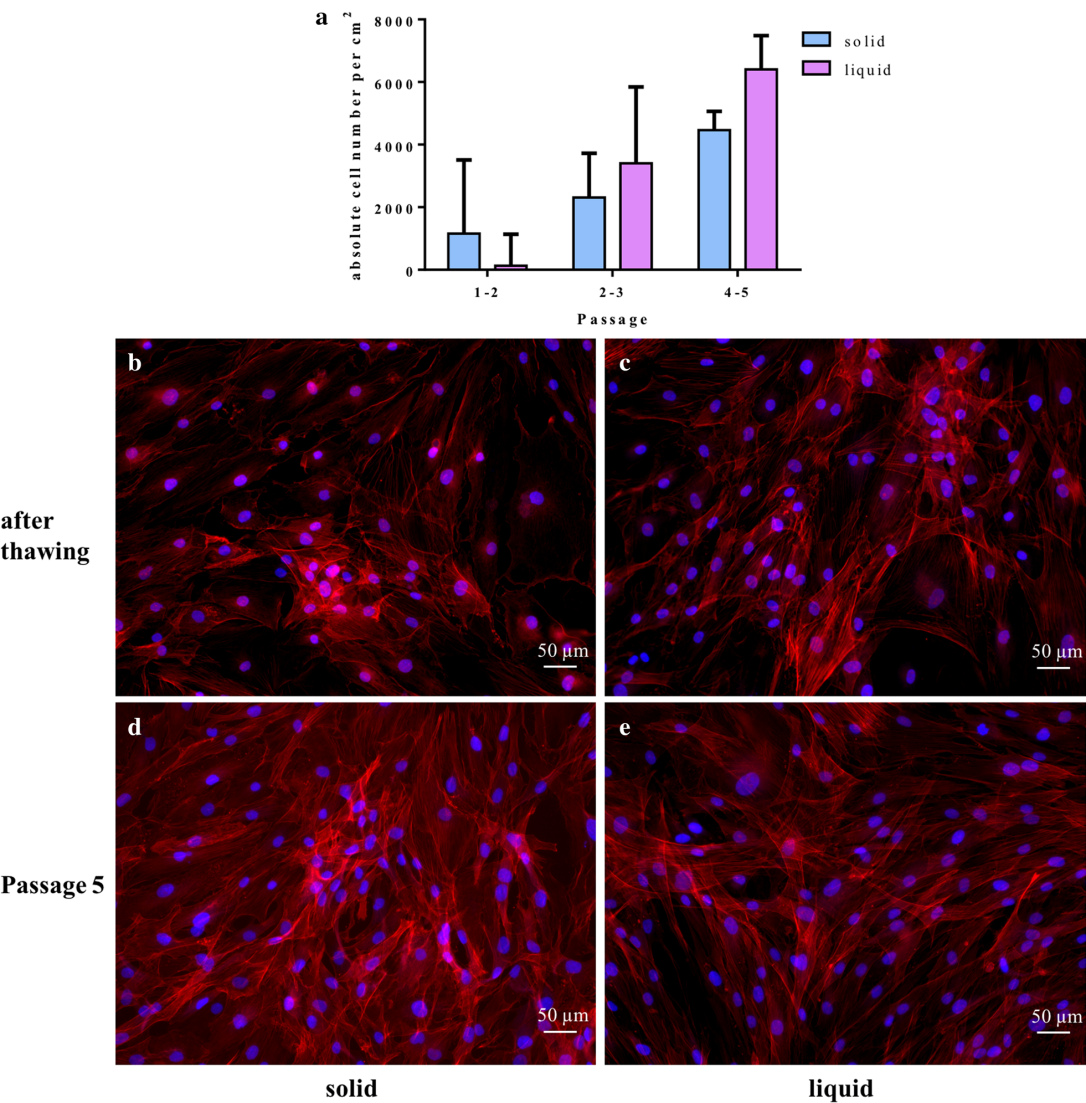
Growth potential (**a**) and cytoskeleton staining (Phalloidin/Hoechst; 200× magnification) of isolated cells from solid adipose tissue after thawing (**b**) and at passage five (**d**) and cells isolated from liquid adipose tissue after thawing (**c**) and at passage five (**e**)

The growth potential for both harvesting procedures increases with a higher passage number (Fig. 6a). The cell numbers’ increase in each passage after 24 h from the liquid fat tissue isolation is minimally higher than the cell numbers’ increase in solid fat tissue. Cells from both harvesting procedures showed increased cell numbers within 24 h after plating with increasing passage number. Apparently, the cells divide faster with each passaging step.

No change of the cytoskeleton could be observed in passage one compared to passage five taking both harvesting procedures into account (Fig. 6b–e).

## Discussion

Mesenchymal stem cells are commonly used in regenerative medicine. Bone marrow is a typical source. It has, however, drawbacks such as infection risk, pain, and fracture risk. Due to this concomitant morbidity, adipose tissue may present a suitable alternative. To obtain adipose tissue, several harvesting techniques exist. It can typically be divided into “liquid” fat (e.g. liposuction procedures) and “solid” fat (e.g. resection of fat tissue). In the literature, not many direct comparisons exist. The total amount of MSCs per volume unit do not differ depending on the harvesting site [10]. However, the total yield from the abdomen seems to be better. However, these authors do not investigate cell differences in association with the harvesting procedure. Another group could show that the abdomen seems to be the best site for obtaining adipose tissue in male patients. This was independent of the chosen harvesting technique [5]. Eto et al. showed that liposuction material lacked large vessels and had significantly more dead cells than resected adipose tissue. The number of stromal cells was about 50% lower in the liposuction compared to the resected tissue [6]. However, these cells were only characterized by negative markers (CD34, CD31, and CD45) and plastic adherence. In our study, similar numbers of MSCs were found independent of the harvesting method.

Liposuction can be performed with different methods such as manual or automated aspiration [14, 16, 18]. There was no difference in MSC characteristics and function comparing manual versus automated procedure [14]. However, some studies report a higher yield and colony formation when using an automated procedure [18]. Automated machines using enzymatic isolation showed an increase in frequency and clonogenic fraction of the adipose-derived MSC [16]. In our study, no changes in growth potential between the two harvesting procedures could be observed. As a matter of fact, they increased their potential in passage to passage up to passage 5.

Purification of MSCs from adipose tissue has been described in different ways. Only lysing erythrocytes is easy, but leads to more impurities with CD34^+^ cells [19]. Comparing enzyme digestion with explant cultures showed higher yields with enzyme digestion than in explant cultures. MSCs characteristics were similar [17, 21]. Enzymatic isolation performed also better compared to mechanical isolation [15]. Taking the above-mentioned positive results into account, we decided to use enzymatic digestion for isolation of MSC from adipose tissue similar to the protocol of Estes et al. [23]. We described it in detail for both lipoaspirates and resected tissue with their specifics and pitfalls.

The isolated MSCs present the typical MSC markers over a long time. Thus, AMSCs can be used as they do not lose their MSC ability. We can conclude that the cells after thawing are not immediately usable for experiments, because the percentage of the positive MSCs markers is very low and the cells need time for regeneration to develop their complete MSC ability. Interestingly, the MSC ability is not lost after thawing and MSC markers reappear within one passage. Minonzio et al. [24] have also shown similar results. Furthermore, cryopreservation of the complete adipose tissue also showed that the MSCs isolated from tissue immediately after thawing did not perform well. However, after 1 passage, no differences with non-frozen tissue could be observed [25].

## Conclusion

We showed that the cells isolated from liquid fat tissue grow faster in higher passages than the cells from solid fat tissue. But both fat tissue types are suitable for experiments. The metabolic activity decreased for both isolation locations. The slopes at passage five are identical. Thus, there is no difference between both isolatable tissue types for further experiments. This is also shown regarding the cytoskeleton staining, where both isolated cells have no change of the structure. Liposuction was performed in the abdominal area, whereas solid fat was obtained from resections in the hip/thigh area. The localization may influence the results as different markers (non-MSC markers) are expressed differently [20]. Others describe no large differences in MSCs derived from different localizations. Subcutaneous fat, as also used by us for both liposuction and resection, showed most stable results [9]. Our method for isolation of AMSCs from resection and liposuction yields similar and good quality MSCs and is thus suitable for regenerative medicine therapies.

## Abbreviations

MSC: mesenchymal stem cells
CD: clusters of differentiation
BM: bone marrow
AMSC: adipose-derived mesenchymal stem cell
DMEM: Dulbecco’s modified eagle medium
FBS: fetal bovine serum
P/S: penicillin/streptomycin
DPBS: Dulbecco’s phosphate buffered saline
DMSO: dimethyl sulfoxide
RT: room temperature
w/o: without
EDTA: ethylenediaminetetraacetic acid
CO_2_: carbon dioxide
